# On the Origin of Shame: Does Shame Emerge From an Evolved Disease-Avoidance Architecture?

**DOI:** 10.3389/fnbeh.2020.00019

**Published:** 2020-02-18

**Authors:** John A. Terrizzi Jr., Natalie J. Shook

**Affiliations:** ^1^Department of Psychology & Philosophy, Texas Woman’s University, Denton, TX, United States; ^2^School of Nursing, University of Connecticut, Storrs, CT, United States

**Keywords:** disgust, shame, behavioral immune system, guilt, evolutionary psychology

## Abstract

Shame and disgust are believed to be evolved psychological solutions to different adaptive challenges. Shame is thought to promote the maintenance of social hierarchies (Gilbert, 1997; Fessler, 2004), whereas disgust is believed to encourage disease avoidance (Curtis et al., 2004; Oaten et al., 2009). Although shame and disgust are often treated as orthogonal emotions, they share some important similarities. Both involve bodily concerns, are described as moral emotions, and encourage avoidance of social interaction. The purpose of the current research was to examine whether shame is uniquely related to disgust and pathogen avoidance. To rule out an association due to the negative valence of both emotions, guilt was also examined. In Study 1, disgust sensitivity and fear of contamination were positively correlated with shame, but not guilt, even after controlling for negative affect. In Study 2, a disgust induction increased shame, but not guilt, for individuals who were sensitive to disgust. The current research provides preliminary evidence for unique relation between shame and disgust.

## Introduction

Traditionally, shame and disgust have been treated as orthogonal emotions. Disgust is described as a basic or primary cross-cultural emotion (Ekman et al., 1987), and shame is discussed as a secondary self-conscious emotion (Tangney et al., 2007). However, some researchers have acknowledged a relation between shame and disgust (see Gilbert, 1998; Nussbaum, 2004; Power and Dalgleish, 2008). Indeed, shame and disgust share several important similarities. Both emotions involve bodily concern, are described as moral emotions, and encourage avoidance of social interaction. The current work took a novel approach to understand the emergence of the self-conscious emotion of shame. More specifically, shame may emerge from an evolved disease avoidance architecture. That is, shame may stem from the primary emotion of disgust being reflected on the self (i.e., perceiving the self as a source of contamination). If so, shame should be uniquely related to disgust and disease avoidant cognitions.

### Shame

Shame is considered broadly as an emotion that involves self-reflection and evaluation (Tangney, 2003). In defining shame, it is important to disentangle it from its sister-emotion, guilt. Although shame and guilt are positively correlated and are often used interchangeably among laypersons, empirical evidence suggests that they are, indeed, different emotional experiences that lead to very different psychological and behavioral outcomes (Tangney, 1991). One important characteristic that distinguishes shame from guilt is the object that is the focus of self-conscious scrutiny (Lewis, 1971). In response to a moral transgression, a person experiencing shame would be likely to think “I am a bad person” whereas someone experiencing guilt would be likely to think “I did a bad thing” (Niedenthal et al., 1994). The experience of shame encourages self-evaluative ruminations that are degrading and pervade all aspects of the self (i.e., both physical and psychological). As such, the self is perceived as innately flawed. Thus, shame is a negatively valenced self-conscious emotion that results in global self-condemnation (Tangney, 1991; Niedenthal et al., 1994).

Shame can be triggered by both moral transgressions and social norm violations (Ferguson et al., 1991; Keltner and Buswell, 1996). For example, Ferguson et al. (1991) demonstrated that imagining scenarios in which one was either responsible for damaging somebody’s property (i.e., moral transgression) or passed gas in public (i.e., norm violation) both elicited shame. Public exposure (i.e., the presence of others) also increases the likelihood of experiencing shame (Smith et al., 2002). If others witness the social norm violation, there is a greater likelihood that the transgressor will experience shame. As such, shame appears to serve an important social function as an internal regulatory system that discourages moral or social norm violations.

In addition to social rules, shame may also be linked to the corporeal, bodily self. Gilbert (1997) suggested that shame is an emergent consequence of the innate human desire to be perceived as attractive. According to Gilbert, attractiveness is one factor that determines relative social standing, and shame is an emotional response to the loss of attractiveness and the accompanying loss of social interaction. In support of this link, evidence suggests that shame plays a key role in disorders that involve body image, such as body dysmorphic disorder (Parker, 2003) and the onset and maintenance of eating disorders (Goss and Allan, 2009).

Finally, shame has also been described as “maladaptive,” because it encourages dysfunctional behaviors, particularly behavioral avoidance (Tangney, 1991; Niedenthal et al., 1994; Orth et al., 2006). For example, when individuals commit a moral transgression, those who are prone to shame are more likely to respond with anger and avoidance rather than empathy and apology, which could repair the damage that is caused by the transgression (Tangney, 1991). The stigma and avoidant behavior that accompany shame may perform a very specific social function. According to Fessler (2004), the function of shame is to regulate social systems and hierarchies. In fact, he speculates that shame is responsible for the aversive effects of social rejection and may ultimately be responsible for encouraging the maintenance of social norms. Indeed, the recollection of childhood social rejection (e.g., ignoring by parents) is associated with chronic shame in adulthood (Claesson and Sohlberg, 2002). Thus, shame may play an integral role in preserving social order (Gilbert, 1997; Fessler, 2004). Similarly, others have suggested that shame may perform an important adaptive function in terms of the maintenance of social norms and moral behavior (Tangney and Stuewig, 2004).

### Disgust

Like shame, disgust is a negative moral emotion that involves bodily concerns and has important implications for social behavior. Darwin (1872) originally referred to disgust as “something revolting, primarily in relation to the sense of taste, as actually perceived or vividly imagined” (p. 254). More recently, disgust has been described as a cross-cultural human emotion (Ekman, 1970) that is an evolved solution to the adaptive challenge of bodily contamination and infectious disease (Curtis and Biran, 2001; Oaten et al., 2009). More specifically, disgust has been described as a disease-avoidance mechanism and a component of the behavioral immune system (BIS; Schaller, 2006; Oaten et al., 2009).

The BIS is a constellation of psychological responses that are evolved solutions to the adaptive challenge of infectious disease (Schaller, 2006). Whereas the function of the biological immune system is to defend the body against pathogens once they have entered the body, the role of the BIS is to encourage the avoidance of situations that could lead to contamination. The BIS promotes prophylactic responses by inducing adaptive affective (e.g., disgust), cognitive (e.g., thoughts of contamination), and behavioral (e.g., avoidance) reactions when individuals are exposed to potentially contaminated stimuli.

Disgust is perhaps the most studied BIS mechanism. It can be conceptualized in terms of a mechanism that can be activated (i.e., turned on or off) by a range of sensory information indicative of contamination, such as the taste of sour milk or the smell of garbage. Because disgust is believed to be an evolved solution to an adaptive challenge, most individuals experience it on at least some level. However, there is significant variability in disgust sensitivity. Thus, like most psychological traits, disgust can be assessed as a personality characteristic. Those who are more sensitive to disgust are overly concerned with potential contamination. They are susceptible to Type I errors (i.e., believing that something is a disease threat when it is not) and are more sensitive to disgusting stimuli. From this perspective, the cost of being too sensitive to disgust is the loss of potentially viable resources due to fear of contamination, whereas the benefit is reduced exposure to infectious disease.

Disgust has been described as a moral emotion concerning purity related social norms (e.g., taboo; Haidt, 2003). Inducing disgust by exposing participants to varying amounts of fart spray (e.g., none, four sprays, or eight sprays) resulted in increased severity of moral judgments (e.g., reactions to eating a dead family dog; Schnall et al., 2008b). Conversely, priming cleanliness (i.e., deactivating disgust and contamination concerns) resulted in less severe moral judgments (Schnall et al., 2008a).

In addition to moral judgments, disgust is also associated with negative attitudes and avoidance of other people. One of the primary vehicles for disease transmission is other human beings. Thus, people who pose a significant disease threat should evoke disgust. Schaller and Duncan (2007) have argued that disgust should encourage individuals to prefer in-group members over outgroup members because outgroup members pose a greater disease threat (i.e., they may harbor pathogens for which ingroup members have no immunity). Indeed, individual differences and activation of disgust have been linked to avoidance of and prejudice toward a wide range of outgroup members including individuals who are foreign, obese, disabled, or sexual minorities (Park et al., 2003, 2006; Faulkner et al., 2004; Navarrete and Fessler, 2006; Olatunji, 2008; Inbar et al., 2009; Terrizzi et al., 2010).

It has also been suggested that disgust may encourage avoidance of potentially contaminated outgroup members by encouraging the formation of socially conservative value systems that promote adherence to social norms and tradition as well as negativity toward and avoidance of outgroup members (Tangney and Stuewig, 2004; Terrizzi et al., 2010). Disgust sensitivity is predictive of a wide range of socially conservative value systems (Terrizzi et al., 2013). Thus, disgust may play an important role in shaping social interactions (e.g., prejudice and avoidance) and constructing social value systems (e.g., social conservatism) that are supportive of those interactions.

### Shame and Disgust

Based on the previous review, there are a number of similarities between shame and disgust. Both emotions appear to play an important role in social interactions, promoting avoidance of others, but for very different purposes (Orth et al., 2006; Oaten et al., 2009). For shame, social avoidance serves to protect the self from the damaging effects of social norm violations (Orth et al., 2006; Schmader and Lickel, 2006; Tangney et al., 2007), whereas, for disgust, social avoidance enables disease avoidance (Faulkner et al., 2004; Navarrete and Fessler, 2006). Indeed, considerable evidence suggests that disgust shapes social interactions *via* the formation of negative attitudes toward other people. Likewise, disgust may play an important role in the formation of attitudes toward the self (e.g., the self-conscious emotion of shame). Further emphasizing the unique relation between shame and disgust, guilt does not share the same pattern of behavioral avoidance. Instead, guilt is characterized by approach behavior (e.g., apologizing; Orth et al., 2006; Schmader and Lickel, 2006).

Shame and disgust seem to also provide similar functions in terms of the maintenance of social norms. Both have been defined as moral emotions, which encourage adherence to social norms and moral behavior (Haidt, 2003; Tangney et al., 2007). Indeed, both encourage moral decision making (Tangney et al., 2007; Schnall et al., 2008a). Furthermore, deficiencies in shame or disgust are associated with psychopathy, which is characterized by an anti-social disregard for social norms (Morrison and Gilbert, 2001; Tangney et al., 2003; Tybur et al., 2009).

Some theorists have argued that shame and disgust are linked in that they both involve bodily or self-condemnation, whereas guilt and anger are emotions that involve condemnation of action or behavior (Roseman, 1984; Nussbaum, 2004). Giner-Sorolla and Espinosa (2011) tested whether exposing participants to the social cues of either disgust or anger would result in increased experience of shame and guilt, respectively. Across two cultures (i.e., the United Kingdom and Spain), participants who were exposed to pictures depicting the facial expression of disgust experienced more shame than guilt, and participants who saw angry faces experienced more guilt than shame.

Another similarity between shame and disgust is the body language and posture that are associated with the two emotions. Darwin (1872) described shame as turning the body away in an attempt to avoid and disgust as pushing away in an attempt to guard the self. Wallbott (1998) found that both shame and disgust involve a collapse of the upper body and downward movement of the head, making the body a smaller target as if to avoid harm. With shame, the postural change may be an attempt to avoid the stigma that accompanies moral transgression, a symbolic attempt to keep the self free of contamination. With disgust, this behavior serves the more practical function of protecting the self from bodily contamination.

Additionally, both shame and disgust share is concerned with the body or the self. The bodily concern that is associated with shame is evinced by its association with body image disorders (Gilbert, 1997; Parker, 2003; Goss and Allan, 2009). Disgust too is concerned with the maintenance of the body in that its primary function is to protect the bodily self from contamination (Oaten et al., 2009). Although shame and guilt are highly related, only shame shares a bodily concern with disgust.

Finally, there is some neuroimaging work that suggests that shame and disgust may have underlying physiological commonalities. Shame has been associated with activation of the anterior cingulate cortex (ACC; Michl et al., 2014). Likewise, disgust has been associated with increased activation of the ACC (Wicker et al., 2003; Amir et al., 2005). Disgust has also been associated with the anterior cingulate gyrus (ACCg; Mataix-Cols et al., 2008), a specific subregion of the ACC which has been linked to processing information related to social interactions (e.g., costs, benefits, errors; Apps et al., 2016). More specifically, individual differences in disgust sensitivity modulate the activation of the ACCg (Mataix-Cols et al., 2008). That is when participants are exposed to a disgusting stimulus, those who are more sensitive to disgust experience more activation in the ACCg. Imaging studies have also shown that both shame and disgust have been associated with activation of the anterior insula (Wicker et al., 2003; Cracco et al., 2016).

One possible explanation for the apparent relation between disgust and shame is that they are overlapping psychological systems. Evolution is a haphazard yet efficient process that takes advantage of existing architecture (Buss et al., 1998; Marcus, 2008). For example, the feather is thought to be an exaptation, a feature that originally evolved to solve one adaptive challenge but was later co-opted to solve another (Buss et al., 1998). The feather provides an important structural function enabling avian flight, but it is thought to have originally evolved as a means of temperature regulation. Much like the feather now serves a different purpose than the one for which it originally evolved, disgust too may serve a different purpose. That is, in addition to its primary role of encouraging disease avoidance, disgust may play an important role in the maintenance of social interactions by evoking shame.

Accordingly, shame may stem, at least in part, from the emotion of disgust. That is, the secondary, self-conscious emotion of shame may be experienced when the primary emotion of disgust is reflected on the self. From this perspective, disgust may serve as an internal moral and social regulatory system in that once a social transgression has been perpetrated, the self is perceived as a source of contamination. The stigmatization that accompanies self-disgust and contamination then serves as an internal contingency that can motivate hiding and avoidance in order to prevent further contamination. In other words, shame may emerge from disgust. As a result of this relation, shame should not only be related to disgust sensitivity, it should also be related to contamination concerns, or disease avoidant cognitions (e.g., Perceived Vulnerability to Disease, PVD).

### Current Research

The goal of the proposed studies was to investigate the role that disgust plays in the self-evaluative emotion of shame. Shame and disgust are thought to have evolved to solve different adaptive challenges (i.e., establishing social hierarchies and disease avoidance, respectively; Gilbert, 1997; Curtis et al., 2004; Fessler, 2004; Oaten et al., 2009). However, the two emotions may be more closely related than previously thought. Both shame and disgust have been described as moral emotions (Haidt, 2003; Tangney and Stuewig, 2004; Schnall et al., 2008b), have similar postural (e.g., shrinking, collapsing, turning away; Darwin, 1872; Wallbott, 1998) and behavioral responses (e.g., avoidance; Tangney, 1991; Oaten et al., 2009), and involve bodily concern (Gilbert, 1997; Parker, 2003; Goss and Allan, 2009; Oaten et al., 2009). Together, these results suggest that disgust and shame may be overlapping systems.

Little research has investigated the association between disgust and shame. Consequently, the goal of the current studies was to investigate the extent to which shame is uniquely related to disgust, as well as disease avoidance concerns more broadly (i.e., contamination concern). Although shame and guilt are often highly correlated (Tangney, 1991), the features that characterize disgust and shame (e.g., bodily concern and social avoidance) do not describe guilt. Therefore, the proposed association between disgust and self-evaluation should specifically result in shame, not general negative self-conscious emotions or guilt. In order to ensure that the relationship is indeed unique to the emotions of disgust and shame, guilt and negative affect were included as comparison and control variables.

If shame and disgust share some evolved psychological architecture in which the experience of shame emerges from perceiving the self as a source of disgust and contamination, disgust sensitivity and disease-avoidant cognitions (e.g., PVD) should predict shame proneness. Additionally, if the effect is specific to shame, disgust sensitivity and disease-avoidant cognitions should not be correlated with guilt and should remain significant even after controlling for negative affect. Furthermore, if shame emerges from disgust, there should be a causal relation between the two systems such that inducing disgust should result in increased shame proneness, but not guilt proneness (i.e., a greater likelihood to respond to prompts of social transgressions with shame consistent responses such as avoidance as opposed to guilt consistent responses such as apologizing). Thus, it was hypothesized that individual differences in disgust sensitivity would be positively correlated with shame, but not guilt, propensity. Moreover, inducing disgust would increase shame, but not guilt, propensity.

## Study 1

The purpose of Study 1 was to investigate whether individual differences in disgust sensitivity and contamination fears were associated with individual differences in shame propensity and sensitivity. If shame emerges from disgust, disgust sensitivity should be positively correlated with shame. Furthermore, shame should be associated with broader disease avoidance concerns, so shame should also be associated with contamination concerns. To ensure that these relations are not mere products of negative affect, we controlled for negative effects in all analyses. Additionally, the relations should be specific to shame (i.e., disgust sensitivity should not be correlated with guilt), so we included guilt-proneness measures to demonstrate discriminant validity.

### Method

#### Participants

There were 195 introductory to psychology students from Virginia Commonwealth University (71% female), who participated in the study for course credit. Participants ranged in age from 18 to 47 years-of-age (*M* = 20.21, *SD* = 3.33). Fifty-one percent of the sample was White, 18% were African-American, 14% were Asian, 4% were Hispanic, and 13% were “Other” or undisclosed.

#### Measures and Procedure

The participants completed a series of questionnaires online in the following order. The questionnaires included measures of disgust sensitivity, contamination concerns, shame and guilt-proneness, mood, and demographic questions.

##### Disgust Measures

General disgust sensitivity was assessed using the Disgust Scale (DS; Haidt et al., 1994; *α* = 0.81), which is a 32-item scale. The first 16 items are scored on a 5-point scale ranging from 0, *strongly disagree*, to 5, *strongly agree* (originally assessed as true/false). The remaining 16 items are scored on a 5-point scale ranging from 0, *not disgusting at all*, to 5, *extremely disgusting* (originally assessed on a 3 point scale). DS scores were computed by taking the average of the 32 items. An example item from the scale is “I might be willing to try eating monkey meat, under some circumstances.”

Pathogen, sexual, and moral disgust sensitivities were measured using the Three Domain Disgust Scale (TDDS; Tybur et al., 2009). The scale contains 21 items measured on a 7-point scale ranging from 0, *not at all disgusting*, to 6, *extremely disgusting*. Scores for each of the subscales were calculated by averaging the subscale items. Example items include “stepping on dog poop” (pathogen, *α* = 0.83), “hearing two strangers have sex” (sexual, *α* = 0.86), and “shoplifting a candy bar from a convenience store” (moral, *α* = 0.89).

The Disgust Propensity and Sensitivity Scale-Revised was used to assess disgust reactivity (DPSS-R; van Overveld et al., 2010; *α* = 0.89). The DPSS-R is a 16-item scale which contains two 8-item subscales: disgust propensity (*α* = 0.78) and disgust sensitivity (*α* = 0.79). The propensity subscale assesses how easily an individual’s disgust reaction is triggered whereas the sensitivity subscale measures the emotional intensity of the reaction. The responses range from 1, *never*, to 5, *always*. DPSS-R scores were calculated by averaging the subscale items. Example items include “I screw up my face in disgust” (Propensity) and “Disgusting things make my stomach turn” (Sensitivity).

##### Contamination Concern

Fear of contamination was assessed using the contamination obsessions and washing compulsions subscale of the Padua Inventory (PI-COWC; Burns et al., 1996; *α* = 0.85). The PI-COWC is a 10-item (e.g., “I find it difficult to touch garbage or dirty things.”) subscale. Participants indicate the extent to which they experience each statement on a scale from 0 (*not at all*) to 4 (*very much*). The PI-COWC score was calculated by averaging the items.

##### Shame and Guilt Measures

The Test of Self-Conscious Affect (TOSCA; Tangney and Dearing, 2002) was used to measure shame and guilt-proneness (*α* = 0.77 and 0.78, respectively). Participants read 15 scenarios (e.g., at work, you wait until the last minute to plan a project and it turns out badly) and rated the extent to which they would respond in a shameful or guilty manner. Item averages were created for the guilt and shame subscales of the TOSCA.

The Guilt and Shame Proneness Scale (GASP; Cohen et al., 2011; *α* = 0.79) is a 20-item scale. Response options range from 1, *very unlikely*, to 7, *very likely*. The GASP contains two 5-item subscales of shame and two 5-item subscales of guilt. The shame subscales include a measure of Negative-Self-Evaluation, which assesses global self-condemnation, and a measure of Withdrawal, which assesses an individual’s desire to avoid contact with other people following a moral or social contract violation. The guilt subscales include a measure of Negative-Behavior-Evaluation, which assesses behavior condemnation, and a measure of Repair, which assesses the likelihood of prosocial behavior following a moral or social contract violation. For each of the subscales, item averages were computed.

##### Control Measure

Mood was controlled for using the Positive and Negative Affect Schedule (PANAS; Watson et al., 1988). The scale is composed of 20 adjectives, 10 positive adjectives (e.g., interested; *α* = 0.90) and 10 negative adjectives (e.g., upset; *α* = 0.87). For each adjective, the participants are asked to rate how much they feel it on a 5-point scale from 1 (*not at all*) to 5 (*extremely*). Item averages were computed for the positive and negative affect subscales. Participants were also asked basic demographic information (e.g., age, sex, ethnicity).

### Results

The means, standard deviations, and reliabilities for all measures are presented in Table 1. Previous research has demonstrated significant sex differences in disgust and shame (Lewis, 1971; Druschel and Sherman, 1999), so males and females in the current sample were compared. Indeed, in the current sample females reported more disgust sensitivity (*M* = 2.85, *SD* = 0.45) than males (*M* = 2.30, *SD* = 0.45), *t*_(174)_ = 7.19, *p* < 0.01, η = 0.23, *d* = 1.22. Females were also higher in shame proneness (*M* = 3.13, *SD* = 0.66) compared to males (*M* = 2.64, *SD* = 0.60), *t*_(181)_ = 4.70, *p* < 0.01, η = 0.11, *d* = 0.78). Given these results, subsequent analyses were conducted with and without sex included as a covariate. Although the correlations were slightly attenuated when sex was controlled, they remained significant and in the predicted direction. Thus, results are reported without sex included as a covariate.

**Table 1:** Means, Standard Deviations, and Cronbach’s Alphas for all Measures in Study 1.

	*M*	*SD*	*α*
DS	2.70	0.52	0.91
TDDS-Pathogen	4.10	1.14	0.88
TDDS-Sexual	3.51	1.44	0.84
TDDS-Moral	3.80	1.38	0.81
DPSS-R	2.68	0.68	0.88
Propensity Subscale	2.88	0.74	0.85
Sensitivity Subscale	2.48	0.80	0.82
PI-COWC	1.52	1.00	0.92
TOSCA-Shame	2.99	0.68	0.84
TOSCA-Guilt	3.98	0.66	0.89
GASP-Shame	4.18	1.00	0.79
NSE Subscale	5.16	1.31	0.83
Withdraw Subscale	3.21	1.20	0.76
GASP-Guilt	5.01	1.12	0.84
NBE Subscale	4.72	1.37	0.79
Repair Subscale	5.33	1.12	0.73
PANAS-Negative Affect	1.71	0.69	0.89
PANAS-Positive Affect	2.67	0.88	0.89

*Note. DS, Disgust Scale; TDDS, Three Domain Disgust Scale; DPSS-R, Disgust Propensity and Sensitivity Scale-Revised; PI-COWC, Checking Obsessions and Washing Compulsions subscale of the Padua Inventory; TOSCA, Test of Self-Conscious Affect; GASP, Guilt and Shame Proneness; NSE, Negative-Self-Evaluation; NBE, Negative-Behavior-Evaluation; PANAS, Positive and Negative Affect Schedule*.

#### Zero-Order Correlations

Zero-order correlations between all measures were calculated to examine general patterns among the constructs (see Table 2). As expected, the measures of disgust sensitivity and contamination concern were consistently positively correlated with the measures of shame (*r*s = 0.13–0.49). Additionally, disgust was positively related to guilt, although these correlations were not consistent across measures. Guilt was consistently correlated only with sexual disgust (*r*s = 0.13–0.38) and moral disgust (*r*s = 0.30–0.36). The shame and guilt measures were highly intercorrelated (*r*s = 0.33 and 0.57). The negative affect was positively correlated with some of the measures of disgust (i.e., the DPSS-R subscales; *r*s = 0.17 and 0.21), but not correlated with other measures (i.e., DS and TDDS; *r*s = 0.01–0.06). The negative affect was not significantly correlated with shame (*r*s = 0.06 and 0.14), but the correlations were in the anticipated directions and some were approaching significance. Unexpectedly, negative affect was negatively correlated with guilt (*r*s = −0.16 and −0.17)^1^. Positive affect was not significantly correlated with any of the measures of disgust (*r*s = −0.01–0.07) or shame (*r*s = −0.05 and −0.10). It was, however, significantly positively correlated with guilt (*r*s = 0.13 and 0.18).

**Table 2:** Zero-Order Correlations for all Measures in Study 1.

	*R*																	
Measure	1	2	3	4	5	6	7	8	9	10	11	12	13	14	15	16	17	18
1. DS		0.65**	0.62**	0.15*	0.44**	0.36**	0.43**	0.62**	0.29**	0.35*	0.14	0.41**	0.16*	0.27**	0.36**	0.10	0.02	0.04
2. TDDS-Pathogen			0.49**	0.17*	0.42**	0.34**	0.39**	0.49**	0.25**	0.27**	0.17*	0.24**	0.11	0.18*	0.19**	0.12	0.01	0.06
3. TDDS-Sexual				0.43**	0.29**	0.13	0.39**	0.48**	0.31**	0.33**	0.21**	0.32**	0.23**	0.29**	0.38**	0.13	0.04	0.01
4. TDDS-Moral					0.18*	0.10	0.23**	0.21**	0.27**	0.32**	0.37**	0.13	0.36**	0.41**	0.43**	0.30**	0.03	0.02
5. DPSS-R						0.88**	0.89**	0.51**	0.45**	0.33**	0.14	0.37**	0.03	0.09	0.13	0.01	0.22**	0.00
6. DPSS-Propensity							0.57**	0.32**	0.31**	0.26**	0.11	0.29**	−0.01	0.03	0.07	−0.03	0.17*	0.04
7. DPSS-Sensitivity								0.56**	0.49**	0.32**	0.14	0.37**	0.06	0.13	0.16*	0.04	0.21**	0.03
8. PI-COWC									0.27**	0.24**	0.08	0.29**	0.04	0.15*	0.24**	0.01	0.04	0.07
9. TOSCA-Shame										0.53**	0.41**	0.43**	0.33**	0.27**	0.32**	0.16*	0.06	−0.10
10. GASP-Shame											0.81**	0.77**	0.38**	0.57**	0.61**	0.41**	0.01	−0.05
11. NSE												0.25**	0.52**	0.68**	0.63**	0.59**	−0.11	0.00
12. Withdraw													0.07	0.20**	0.31**	0.02	0.14	−0.07
13. TOSCA-Guilt														0.63**	0.56**	0.58**	−0.17*	0.13
14. GASP-Guilt															0.92**	0.88**	−0.16*	0.18*
15. NBE																0.61**	−0.15*	0.15*
16. Repair																	−0.14*	0.18*
17. Negative Affect																		0.02
18. Positive Affect																		

Note. *p < 0.05, **p < 0.01. DS, Disgust Scale; TDDS, Three Domain Disgust Scale; TOSCA, Test of Self-Conscious Affect; GASP, Guilt and Shame Proneness; NSE, Negative-Self-Evaluation; NBE, Negative-Behavior-Evaluation.

#### Partial Correlations

In order to ensure that the relation between shame and disgust was specific to shame, guilt and negative affect were partially out of the correlations. Additionally, separate analyses were conducted to examine the relation between guilt and disgust. For those analyses, both shame and negative affect were partially out of the correlations. The partial correlations are presented in Table 3^2^.

**Table 3:** Partial Correlations of Shame and Guilt with Disgust and Contamination Concern for Study 1.

	Shame^1^					Guilt^2^				
	TOSCA	GASP	NSE	Withdraw	Composite	TOSCA	GASP	NBE	Repair	Composite
DS	0.27**	0.27**	−0.01	0.40**	0.31**	0.02	0.09	0.20*	−0.06	0.06
TDDS-Pathogen	0.25**	0.24**	0.12	0.25**	0.28**	−0.01	0.00	−0.01	0.00	−0.01
TDDS-Sexual	0.28**	0.24**	0.03	0.31**	0.30**	0.12	0.14	0.23**	0.01	0.15
TDDS-Moral	0.12	0.10	0.11	0.05	0.13	0.30**	0.32**	0.34**	0.22**	0.35**
DPSS-R	0.47**	0.34**	0.13	0.37**	0.47**	−0.12	−0.16	−0.12	−0.15	−0.16
Propensity	0.33**	0.26**	0.12	0.26**	0.35**	−0.08	−0.11	−0.08	−0.11	−0.11
Sensitivity	0.51**	0.35**	0.11	0.40**	0.50**	−0.13	−0.17*	−0.14	−0.16*	−0.17*
PI-COWC	0.29**	0.28**	0.07	0.34**	0.33**	−0.14	−0.06	0.05	−0.17*	−0.11

Note. *p < 0.05, **p < 0.01. DS, Disgust Scale; TDDS, Three Domain Disgust Scale; DPSS-R, Disgust Propensity and Sensitivity Scale-Revised; PI-COWC, Checking Obsessions and Washing Compulsions subscale of the Padua Inventory; TOSCA, Test of Self-Conscious Affect; GASP, Guilt and Shame Proneness; NSE, Negative-Self-Evaluation; NBE, Negative-Behavior-Evaluation. ^1^Controlling for guilt and negative affect. ^2^Controlling for shame and negative affect.

As hypothesized, disgust sensitivity and contamination concern were positively correlated with shame even after controlling for guilt and negative affect. The only measure of disgust that was not correlated with shame was moral disgust. These results suggest that the relation between disgust and shame involves physical or bodily contamination rather than symbolic moral contamination. Interestingly, the Negative Self-Evaluation subscale of the GASP, which assesses global self-condemnation, was the only indicator of shame that was not significantly correlated with disgust. On the other hand, disgust was strongly correlated with the Withdraw subscale, which assesses an individual’s desire to avoid situations that could induce shame.

Guilt was not consistently correlated with the disease-avoidance components of disgust (i.e., core/pathogen and sexual disgust) when controlling for shame and negative affect. However, guilt was consistently positively correlated with moral disgust. Although this finding was not hypothesized, it is consistent with the literature which describes guilt as a moral emotion that is concerned with social contract violations (Tangney et al., 2007).

### Discussion

Study 1 provided initial correlational evidence that there is a unique relation between disgust and shame. Disgust sensitivity and contamination concern were consistently positively correlated with shame propensity even after controlling for guilt propensity and negative affect. Guilt, on the other hand, was generally not correlated with the disease-avoidant components of disgust when controlling for shame and negative affect. These findings provided partial evidence that shame may involve disgust. Nevertheless, Study 1 was limited in that it was a correlational design and therefore unable to provide any evidence for a causal relation between disgust and shame.

Additionally, Study 1 provided some evidence that moral disgust as opposed to the other disgust sensitivity measures (i.e., pathogen/core disgust and sexual disgust) was consistently positively correlated with guilt propensity even after controlling for shame propensity and negative affect. Interestingly, moral disgust was the only measure of disgust sensitivity that was not correlated with shame propensity. This finding is consistent with the primary distinction between shame and guilt (i.e., that shame is characterized by self-evaluation whereas guilt is characterized by behavioral-evaluation; Niedenthal et al., 1994). As moral disgust is a behavioral-evaluation (i.e., how disgusted an individual is by social contract violations), it makes sense that moral disgust was correlated with guilt-proneness but not shame proneness.

## Study 2

The purpose of Study 2 was to test a causal model in which inducing disgust results in higher levels of shame proneness (i.e., a greater likelihood of responding to prompts of social transgressions with shame consistent responses). If shame is an emotional experience that emerges from feeling disgusted with the self, inducing disgust should trigger shame. Again, the effect was hypothesized to be specific to shame, so the disgust induction was not expected to affect feelings of guilt. Moreover, the effect was expected to be specific to disgust and not the result of general negative affect. Thus, it was hypothesized that inducing disgust would result in higher levels of shame than both a neutral condition and a condition in which a negative mood state was induced. Additionally, as individuals who are sensitive to disgust are presumably more receptive to the disgust manipulation than those who are less sensitive to disgust (see Terrizzi et al., 2010), moderation analyses were conducted. It was hypothesized that those who were sensitive to disgust would experience more shame following the disgust manipulation compared to those who were less sensitive to disgust.

### Method

#### Participants

There were 175 introductory to psychology students from Virginia Commonwealth University (62% female) who received course credit for their participation. They ranged in age from 18 to 41 years-of-age (*M* = 19.29, *SD* = 2.61). Forty-seven percent of the sample was White, 25% were African-American, 17% were Asian, 6% were Hispanic, and 5% were “Other” or undisclosed.

#### Measures and Procedure

Upon arriving at the lab, participants provided informed consent. Next, participants were randomly assigned to either the disgust, negative, or neutral condition and completed the mood induction task, which was masked as a lexical decision task. Following the mood induction, the participants completed the same battery of questionnaires that was used in Study 1, with the addition of the PVD scale (Duncan et al., 2009) as a second measure of contamination concern. First, participants completed the measures of shame and guilt, followed by the disgust and contamination measures, then the PANAS, and lastly the demographic questions. Finally, the participants were debriefed, given credit, and thanked for their participation.

##### Mood Induction

To induce the different mood states, a subliminal priming procedure was utilized through a lexical decision task. Previous research has demonstrated that this is an effective and unobtrusive methodology for priming affective states (e.g., Ferré and Sánchez-Casas, 2014). The participants were introduced to the lexical decision task as a word game. They were told that the purpose of the word game was to assess their ability to recognize words. They were presented with strings of letters and asked to indicate as quickly as possible whether each string of letters was a word or nonword. Prior to each letter string, participants were subliminally primed with either neutral, negative, or disgusting words.

Following the procedure used by Dijksterhuis et al. (2008), each trial included a 50 ms pre-mask (XXXXXX), a 17 ms prime, a 50 ms post-mask (XXXXXX), and the target string of letters. For half of the trials, the target word was a random string of letters (e.g., “tsers”). The remaining trials contained a neutral word (e.g., book). Participants were randomly assigned to one of three conditions (i.e., disgust, negative, or neutral). Each condition contained 10 primes and each prime was repeated five times for a total of 50 trials.

The priming words were matched as closely as possible for length (i.e., number of letters) and starting letter. The priming words for the disgust condition were chosen based on the cross-cultural elicitors of disgust (see Curtis et al., 2004). For the disgust condition, participants were primed with words that evoke bodily disgust (e.g., diarrhea, urine). The participants in the negative condition were primed with words that evoke negativity (e.g., disappointing, useless). Finally, those in the neutral condition were primed with words that do not evoke an emotional response (e.g., door, unit; see Appendix for complete list of primes).

##### Disgust Measures

Since disgust sensitivity has been shown to predict reactivity to disgusting stimuli (van Overveld et al., 2010), there was reason to believe that the efficacy of the disgust manipulation might depend on individual differences in disgust sensitivity and contamination concerns. Thus, participants completed the DS (Haidt et al., 1994) and the TDDS (Tybur et al., 2009), which were used in Study 1, to assess disgust sensitivity.

##### Contamination Concern

The PVD scale was used to assess germ aversion (*α* = 0.74) and perceived infectability (*>α* = 0.87; Duncan et al., 2009). The scale contains two subscales, an 8-item measure of germ aversion (e.g., “I prefer to wash my hands pretty soon after shanking someone’s hand.”) and a 7-item measure of perceived infectability (e.g., “If an illness is ‘going around,’ I will get it”). Participants were asked to respond to items on a 7-point scale from 1 (*strongly disagree*) to 7 (*strongly agree*). Item averages were calculated for the perceived infectability and germ aversion subscales.

##### Shame and Guilt Measures

Shame and guilt were assessed using the same measures as Study 1, the TOSCA (Tangney and Dearing, 2002) and the GASP (Cohen et al., 2011).

##### Control Measures

Mood was controlled for using the PANAS (Watson et al., 1988). Finally, participants were asked basic demographic information (e.g., age, sex, ethnicity).

### Results

The means, standard deviations, and reliabilities for all measures are presented in Table 4. To ensure that the manipulation did not affect disgust sensitivity or mood, three one-way ANOVAs were conducted to compare levels of disgust sensitivity, negative mood, and positive mood among the three conditions. The mood manipulation did not affect trait level disgust sensitivity (*F*_(2,172)_ = 0.28, *p* = 0.75), negative affect (*F*_(2,172)_ = 0.02, *p* = 0.93), or positive affect (*F*_(2,172)_ = 0.73, *p* = 0.48). All subsequent analyses were conducted with and without sex included as a covariate. However, the pattern of results remained the same and significant even after controlling for sex. Thus, analyses are reported without sex included as a covariate.

**Table 4:** Means, Standard Deviations, and Cronbach’s Alphas for all Measures in Study 2.

	*M*	*SD*	*α*
DS	2.69	0.50	0.89
TDDS-Pathogen	4.12	1.14	0.84
TDDS-Sexual	3.46	1.45	0.84
TDDS-Moral	3.80	1.32	0.87
PVD			
Germ Aversion	3.90	1.21	0.71
Perceived Infectability	3.23	1.37	0.84
TOSCA-Shame	2.90	0.64	0.80
TOSCA-Guilt	4.00	0.51	0.77
GASP-Shame	4.09	0.92	0.72
NSE Subscale	5.21	1.21	0.69
Withdraw Subscale	2.97	1.03	0.61
GASP-Guilt	5.18	0.98	0.79
NBE Subscale	4.77	1.30	0.74
Repair Subscale	5.59	0.88	0.56
PANAS-Negative Affect	1.46	0.55	0.84
PANAS-Positive Affect	2.65	0.96	0.91

*Note. DS, Disgust Scale; TDDS, Three Domain Disgust Scale; PVD, Perceived Vulnerability to Disease; TOSCA, Test of Self-Conscious Affect; GASP, Guilt and Shame Proneness; NSE, Negative-Self-Evaluation; NBE, Negative-Behavior-Evaluation; PANAS, Positive and Negative Affect Schedule*.

#### Primary Analyses

The data were analyzed using hierarchical multiple regression following the procedure outlined by Aiken and West (1991). As there were multiple, highly correlated indicators of disgust sensitivity, shame, and guilt, composite variables were created for each of the constructs^3^. The disgust sensitivity composite was created by standardizing and averaging all of the disease-avoidance components of disgust and contamination concern (i.e., DS, TDDS-Pathogen, TDDS-Sexual, and the Germ Aversion subscale of the PVD; *r*s = 0.36–0.69)^4^. Likewise, indexes of shame and guilt were created by standardizing and averaging their respective subscales from the TOSCA and GASP (*r* = 0.65 for shame measures, *r* = 0.73 for guilt measures). Two dummy coded condition variables were created following the steps outlined in Aiken and West (1991). For the first dummy coded variable, the disgust and negative conditions were coded as 0 and the neutral condition was coded as 1, which tested the main effect of the disgust manipulation relative to the neutral condition. For the second dummy coded variable, the disgust and neutral conditions were coded as 0 and the negative condition was coded as 1, which tested the main effect of the disgust manipulation relative to the negative condition. As both of these dummies coded variables shared the contrast between the negative and neutral conditions, that effect was partialled out.

In order to ensure that the effect was not due to a general state of negative mood, the negative affect subscale of the PANAS was entered in the first step of the regression model. For analyses involving shame as the dependent variable, guilt was also entered in the first step as a covariate. For analyses involving guilt as a dependent variable, shame was entered in the first step as a covariate. The dummy coded condition variables and the disgust sensitivity composite were entered in the second step of the analysis^5^. Finally, interaction terms between the condition and the disgust sensitivity composite variables were created by standardizing the disgust sensitivity variable and multiplying it by each condition variable. These interaction terms were added to the third step of the hierarchical regression model.

#### Shame as the Dependent Variable

**Figure 1:**
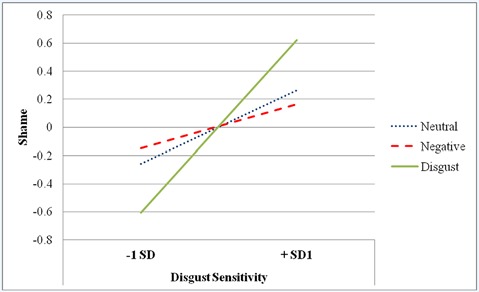
Condition by Disgust Sensitivity Interaction predicting Shame in Study 2.

In Step 1, both of the control variables, negative affect [*β* = 0.17, *p* = 0.02, 95% CI (0.02, 0.31)] and guilt [*β* = 0.54, *p* < 0.05, 95% CI (0.42, 0.64)] emerged as significant predictors of shame. In Step 2, the disgust sensitivity composite was a significant predictor of shame [*β* = 0.15, *p* = 0.03, 95% CI (0.00,0.29)], replicating the effect found in Study 1. There was, however, no main effect for condition. Participants in the disgust condition did not experience more shame than participants in either the neutral condition [*β* = −0.01, *p* = 0.94, 95% CI (−0.16, 0.14)] or the negative condition [*β* = −0.03, *p* = 0.64, 95% CI (−0.18, 0.12)]. However, in Step 3, there was a significant interaction between the disgust sensitivity composite and condition (*R*^2^ *change* = 0.03). More specifically, when comparing the disgust condition to the neutral condition, there was no significant interaction [*β* = −0.13, *p* = 0.12, 95% CI (−0.28, 0.02)]. When comparing the disgust condition to the negative condition, the interaction was significant [*β* = −0.21, *p* = 0.01, 95% CI (−0.35, −0.06)]. The interaction is displayed in Figure 1.

Simple slopes analyses indicated that at high levels of disgust sensitivity (i.e., +1 *SD*), the disgust manipulation did not result in significantly higher levels of shame when compared to the neutral condition [*β* = 0.14, *p* = 0.20, 95% CI (−0.01, 0.29)]. However, at high levels of disgust sensitivity, the disgust manipulation led to significantly higher levels of shame compared to the negative condition [*β* = 0.25, *p* = 0.02, 95% CI (0.10, 0.39)]. At low levels of disgust sensitivity (i.e., −1 *SD*), the disgust manipulation did not have a significant effect on shame compared to the neutral condition [*β* = −0.13, *p* = 0.25, 95% CI (−0.28, 0.02)] or the negative condition [*β* = −0.19, *p* = 0.09, 95% CI (−0.33, −0.04)]. Furthermore, disgust sensitivity significantly predicted shame for participants in the disgust condition [*β* = 0.39, *p* < 0.01, 95% CI (0.25, 0.51)], but did not predict shame levels for participants in the neutral [*β* = 0.08, *p* = 0.47, 95% CI (−0.07, 0.23)] or negative [*β* = −0.07, *p* = 0.60, 95% CI (−0.22, 0.08)] conditions. Thus, for those who were more sensitive to disgust, the disgust manipulation increased shame.

#### Guilt as the Dependent Variable

In Step 1, shame [*β* = 0.56, *p* < 0.01, 95% CI (0.45, 0.66)], but not negative affect [*β* = −0.03, *p* = 0.69, 95% CI (−0.18, 0.12)], emerged as a significant predictor of guilt. In contrast to Study 1, in Step 2, the disgust sensitivity composite remained a significant predictor of guilt even after controlling for shame and negative affect [*β* = 0.17, *p* = 0.01, 95% CI (0.02, 0.31)]. There was, however, no main effect for condition. Participants in the disgust condition did not experience more guilt than participants in either the neutral [*β* = 0.00, *p* = 0.99, 95% CI (−0.15, 0.15)] or the negative condition [*β* = 0.06, *p* = 0.40, 95% CI (−0.09, 0.21)]. In Step 3, the interaction between condition and disgust sensitivity was also not significant when the disgust condition was compared to the neutral condition [*β* = −0.10, *p* = 0.27, 95% CI (−0.25, 0.05)] or the negative condition [*β* = 0.01, *p* = 0.91, 95% CI (−0.14, 0.16)].

### Discussion

As in Study 1, disgust sensitivity was a significant predictor of shame even after controlling for guilt and negative affect. However, unlike Study 1, disgust sensitivity was a significant predictor of guilt even after controlling for shame and negative affect. Although there was no evidence for a main effect of the disgust manipulation, Study 2 provided initial evidence that inducing disgust increased shame for individuals who were sensitive to disgust. These results were significant even after controlling for negative affect and guilt. Moreover, the effect seemed to be particular to shame and disgust. When guilt was analyzed as the dependent variable, there was no interaction between condition and disgust sensitivity when controlling for shame and negative affect. Thus, the results highlight the unique relationship between shame and disgust.

## General Discussion

Across both studies, shame was positively correlated with both disgust sensitivity and contamination concerns (i.e., those who were sensitive to disgust and/or concerned with contamination were more sensitive to shame). More importantly, in both studies, disgust sensitivity and contamination concern were significant predictors of shame even after controlling for guilt and negative affect, emphasizing that the relation between disgust and shame is unique. That is, the relation between disgust and shame was not due to negative affect (i.e., that they are both negatively valenced emotions) and the same pattern was not seen with shame’s sibling emotion, guilt.

Interestingly, however, guilt was consistently positively correlated with moral disgust even after controlling for shame and negative affect. This effect may be due to the fact that shame and guilt differ in regard to the nature of their self-conscious evaluations. For shame, the self is the object of the negative evaluations whereas for guilt, the behavior serves as the attitude-object (Niedenthal et al., 1994). Thus, guilt may be associated with moral disgust because it concerns negative behavioral evaluations (i.e., being disgusted by social contract violations).

Study 2 provided some initial support for a causal relation between disgust and shame. Although there was no main effect for the disgust induction (i.e., inducing disgust did not increase shame for all participants), relative to the negative induction, the disgust manipulation increased shame for individuals who were more sensitive to disgust. Importantly, this effect was consistent even after controlling for negative affect and guilt. Moreover, the manipulation did not have the same effect on guilt (i.e., inducing disgust did not affect guilt).

As a whole, these studies provide some preliminary evidence for a unique relation between shame and disgust. That is, shame may piggyback on evolved disease avoidance architecture. But, it is clear that much more work needs to be done in order to elucidate the exact nature of the relationship between shame and disgust.

### Limitations and Future Directions

One primary limitation of the current studies is that disgust with the self was not directly manipulated. Study 2 induced disgust, but did not directly link disgust with self (i.e., it was not clear that the self was the object of the disgust). Furthermore, it is impossible to rule out participant bias and demand characteristics as potential explanations for the findings in Study 2. As we did not include a pretest measure of shame, it is also impossible to rule out preexisting differences. Future studies should include pretest measures and evaluate whether manipulating disgust toward the self (e.g., having participants imagine or recall scenarios in which they got sick in public) results in more consistent findings. If shame is experienced as disgust with the self, manipulations that evoke disgust with self should be more likely to consistently increase shame.

Additionally, all of the studies assessed shame using explicit measures. Social desirability can be a problem particularly for self-report measures of attitudes toward the self, because people tend to engage in positive illusions (i.e., presenting the self in a more positive light; Heatherton and Wyland, 2003; Oakes et al., 2008). As shame is concerned with negative self-evaluations, this positivity bias could be a problem for the current research because it may make individuals less likely to report shame. Thus, the incorporation of implicit measures or objective physiological measures (e.g., functional magnetic resonance imaging, fMRI; electroencephalography, EEG) may provide a more accurate assessment of shame. Moreover, implicit measures could be used to assess the extent to which individuals associate disgust with the self. If shame is experienced as disgust with the self, individuals who are sensitive to shame should categorize disgusting adjectives (e.g., repulsive) more quickly than negative adjectives (e.g., unpleasant) following self primes (e.g., I or me).

Lastly, although the current studies presented consistent positive correlations between shame and disgust sensitivity and demonstrate that these correlations remain significant even after controlling for negative affect and guilt, it is still possible that the relations may be explained by an unmeasured third variable (e.g., behavioral inhibition or neuroticism). Both disgust and shame have been correlated with behavioral inhibition or behavioral avoidance and neuroticism (Orth et al., 2006; Olatunji et al., 2008; Cohen et al., 2011). Moreover, in the current studies, we controlled for state level, rather than trait level, negative affect. Perhaps the trait level affect could provide a potential alternative explanation. Thus, future research should evaluate whether the relation between disgust and shame persists even after controlling for behavioral inhibition, neuroticism, and trait level negative affect.

## Conclusion

The results from the current research provide some preliminary evidence for a link between shame and disgust. Though additional work needs to be done in order to understand the causal nature of this relation, it may have some important clinical implications for clients who suffer from shame-related psychological disorders (e.g., body dysmorphic disorder and eating disorders). Indeed, given the accumulation of evidence linking disgust sensitivity with anxiety disorders (see Olatunji and McKay, 2009), some researchers have already proposed reducing disgust sensitivity as a component of psychotherapy (Viar-Paxton and Olatunji, 2012). If future experimental evidence validates the causal relation between disgust and shame (i.e., if disgust causes shame), these types of disgust reduction therapies may prove to be effective treatments for shame-related psychological disorders. Additionally, if shame is experienced as disgust with the self, it may help shed light on broader issues such as stigmatization (i.e., stigma may be experienced as self-contamination). What is clear, however, is that more research needs to be done to clarify the relation between disgust and shame.

## Data Availability Statement

The datasets generated for this study are available on request to the corresponding author.

## Ethics Statement

The studies involving human participants were reviewed and approved by Virginia Commonwealth University IRB. The patients/participants provided their written informed consent to participate in this study.

## Author Contributions

Both authors made substantial contributions to the framework of the study and both authors were involved in writing the manuscript.

## Conflict of Interest

The authors declare that the research was conducted in the absence of any commercial or financial relationships that could be construed as a potential conflict of interest.

## Figures and Tables

**Table:** Primes for Study 2.

Disgust	Negative	Neutral
Pus	Pity	Plain
Fart	Fault	Farm
Scab	Scar	Square
Snot	Snob	Street
Puke	Pinch	Plant
Poop	Penalty	Paper
Urine	Useless	Unit
Vomit	Vandal	Vest
Mucus	Maniac	Month
Diarrhea	Disappointing	Door

## Footnotes

^1^These results appear to be an anomaly. In Study 2 and other studies from this lab, guilt was either positively correlated with negative affect or not significantly correlated, as has been found in previous research (Cohen et al., 2011).

^2^Separate analyses were conducted controlling for guilt and negative affect. Both guilt and negative affect marginally attenuated the relation between disgust and shame. For ease of presentation, only the partial correlations in which both guilt and negative affect were controlled simultaneously are presented here.

^3^Separate analyses were conducted for each of the measures of shame, guilt, and disgust sensitivity. As the results were relatively consistent across measures, only the analyses with the composite variables are presented.

^4^Separate analyses were conducted for each of the scales used in the disgust sensitivity composite (i.e., DS, TDDS-Pathogen, TDDS-Sexual, and PVD-Germ Aversion). As the measures were highly correlated and separate analyses exhibited comparable results, only the analyses for the composite measures are presented.

^5^Prior to running the regression analyses, Levene’s test for equality of variances revealed no significant differences across conditions.
